# Dirty Money: A Matter of Bacterial Survival, Adherence, and Toxicity

**DOI:** 10.3390/microorganisms4040042

**Published:** 2016-11-23

**Authors:** Frank Vriesekoop, Jing Chen, Jenna Oldaker, Flavien Besnard, Reece Smith, William Leversha, Cheralee Smith-Arnold, Julie Worrall, Emily Rufray, Qipeng Yuan, Hao Liang, Amalia Scannell, Cryn Russell

**Affiliations:** 1 Department of Food Science and Agri-food Supply Chain Management, Harper Adams University, Newport TF10 8NB, UK; flbesnard.biotech@gmail.com (F.B.); reecesmith345@gmail.com (R.S.); 2 School of Science and Engineering, Federation University, Ballarat 3353, Australia; chenjing1983@gmail.com (J.C.); j_oldaker_3@hotmail.com (J.O.); wleversha@bigpond.com (W.L.); Cheralee.Smith-Arnold@sjog.org.au (C.S.-A.); j.worrall@federation.edu.au (J.W.); yuanqp@mail.buct.edu.cn (Q.Y.); 3 School of Life Sciences, Heriot Watt University, Edinburgh EH14 4AS, UK; rufrayemilie@yahoo.ca; 4 College of Life Science and Technology, Beijing University of Chemical Technology, Beijing 100029, China; starslh@163.com; 5 College of Life Sciences, University College Dublin, Dublin D04 V1W8, Ireland; amalia.scannell@ucd.ie; 6 Faculty of Science and Technology, Eastern Institute of Technology, Taradale 4112, New Zealand; CRussell@eit.ac.nz

**Keywords:** banknotes, bacteria, substrate, coins, microcosm, microbiome

## Abstract

In this study we report the underlying reasons to why bacteria are present on banknotes and coins. Despite the use of credit cards, mobile phone apps, near-field-communication systems, and cryptocurrencies such as bitcoins which are replacing the use of hard currencies, cash exchanges still make up a significant means of exchange for a wide range of purchases. The literature is awash with data that highlights that both coins and banknotes are frequently identified as fomites for a wide range of microorganisms. However, most of these publications fail to provide any insight into the extent to which bacteria adhere and persist on money. We treated the various currencies used in this study as microcosms, and the bacterial loading from human hands as the corresponding microbiome. We show that the substrate from which banknotes are produced have a significant influence on both the survival and adherence of bacteria to banknotes. Smooth, polymer surfaces provide a poor means of adherence and survival, while coarser and more fibrous surfaces provide strong bacterial adherence and an environment to survive on. Coins were found to be strongly inhibitory to bacteria with a relatively rapid decline in survival on almost all coin surfaces tested. The inhibitory influence of coins was demonstrated through the use of antimicrobial disks made from coins. Despite the toxic effects of coins on many bacteria, bacteria do have the ability to adapt to the presence of coins in their environment which goes some way to explain the persistent presence of low levels of bacteria on coins in circulation.

## 1. Introduction

Money is the most recognisable exchange matter with a secure value that is used by people to obtain goods and services, or to accumulate as a safe gathering of wealth. Notwithstanding a hastily moving society where credit cards, mobile phone apps, near-field-communication systems, and cryptocurrencies such as bitcoins are rapidly replacing the use of hard currencies [1,2], cash exchanges still make up a significant means of exchange for small value purchases, while cash is still commonly used in developing countries for high value purchases. Apart from the want of people to possess money, there is a very large body of research that points out that currency is covered in all sorts of filth and microorganisms (e.g., [3,4,5,6,7,8,9]). A search of available literature uncovered in excess of 100 publications that report on the presence of microorganisms on banknotes and/or coins since the late 1800s, with roughly two thirds of those papers being published in the last decade. This considerable interest in the hygienic status of currency predominantly focuses on the isolation of bacteria, and sometimes fungi. However, the interest was most commonly focused on the presence of potential pathogenic microorganisms. It is true that money (banknotes and coins) have all the hallmarks of the ultimate fomite, with repetitive touching of its surface by multiple persons in succession with no real intent or opportunity to clean and/or sanitise. In this sense money is a bit like public phones [10], balustrades in train stations, public transport [11], and sport and playground equipment [12]; however, it could be taken as common sense that materials that are shared around very frequently in public are likely to act as fomites.

The very large volume of publications that describe the presence of bacteria on banknotes and coins fails to provide any insight into the extent to which bacteria adhere and persist on money. Rather than focusing on the occurrence of potentially pathogenic microorganisms on currency per se, we focused on currency (both banknotes and coins) as a microcosm (an environment that support a heterogeneous population of microorganisms that are defined by their typical usage). Hence, we considered both banknotes and coins as microcosms from an experimental point of view. Microcosms represent simplified physical ecosystems used to simulate and predict the behaviour of natural environments, but under controlled conditions. Both banknotes and coins are predominantly handled by multiple humans through touch. The overall human skin microbiome is arguably dominated by four main bacterial phyla: Firmicutes, Actinobacteria, Proteobacteria, and Bacteroidetes [13,14,15], while the hand microbiome is dominated by the Proteobacteria and Bacteroidetes [13,15]. The microbiota of hands can be divided into two groups: firstly, there are the resident microorganisms, which represent a relatively fixed group of microorganisms whose makeup changes very little and tend to recover rapidly following perturbation. Secondly, there is a group of transient microorganisms that rarely establish a permanent residency on the skin but might exist because of external environmental exposures that might persist for a few hours up to a couple of days [15]. We therefore argue that the most likely source of the microorganisms on currency originates from humans [13,14,15,16,17,18] and could be considered as a heterogeneous microbiome.

## 2. Materials and Methods

### 2.1. Sources of Currency Used in This Study

All currency used in the survival and adherence study were taken out of common circulation. All currency was used, but non-damaged. Australian twenty cent “silver” coins and one dollar “gold” coins were used to represent the two most commonly used coin alloys: consisting of 75% copper and 25% nickel, and 92% copper and 2% nickel and 6% aluminium, respectively. Chinese one-yuan or the British five-pound bank notes were used as typical representatives of 100% cotton-based banknotes; one-dollar bills from the USA were used as typical representatives of banknotes made from a cotton-linen blend; the Australian dollar bills were used as representatives of plastic-based banknotes (biaxially oriented polypropylene); and the Japanese 1000 yen notes were used as representatives of washi-style paper banknotes. Approximate details of banknotes are provided in Table 1.

### 2.2. Microbiome Preparation and Inoculation

In order for the bacterial loading on coins and banknotes used in the survival and adherences studies to be realistic and reflective of real-life conditions, we defined the currency microbiome as the bacteria transferred from human hands, as such we sourced our inoculum used in most of the experiments described in this work from the hands of multiple individuals. This was facilitated either by direct touch by human hands, or by soaking the currency in nutrient broth (Oxoid: (g/L) Lab-Lemco (10); Peptone (10); NaCl (5)). When currency was inoculated by direct human contact, at least five volunteers were used to handle the currency continuously for at least 15 min. The volunteers were instructed not to wash their hands prior to their handling of the currency (inoculation). A number of preliminary experiments in which volunteers handled banknotes for inoculation purposes yielded poor and inconsistent results. Since the aim of these experiments was not to identify the makeup of the hand microbiome itself, but instead to determine the survival and adherence of a hand microbiome on currency, we opted for the following procedure. When currency was inoculated by soaking in nutrient broth, at least two volunteers were asked to “wash” their hands in sterile nutrient broth and thoroughly mix the currency for at least 5 min before the currency/nutrient broth mixture was incubated overnight under ambient conditions. Following overnight incubation the currency was allowed to air-dry. Air-dried coins were bagged, three at a time, in sterile plastic bags. Banknotes were placed on a slotted rack to air-dry overnight after which they were placed in a Thermoline Environmental Control Incubator (TRISLH-495-1-SD) at 20 °C and 80% relative humidity. This means that the first quantitative analyses of the bacterial microbiome on the banknotes was carried out at least 18 h following the removal of the banknotes from the inoculation broth.

### 2.3. Bacterial Cultures Used

In this work we evaluated the survival of the following bacteria; *Escherichia coli* O157:H7-VT (N)—NCTC 12900, *Listeria monocytogenes* ATCC 7644, *Salmonella* Typhimurium (kindly provided by the University of Melbourne, Melbourne, Australia), *Staphylococcus aureus* (kindly provided by Victoria University, Melbourne, Australia), and *Cronobacter sakazakii* ATCC 11467 (kindly donated by University College Dublin, Dublin, Ireland). Microorganisms were subcultured weekly at 37 °C, and subsequently maintained at 4 °C on nutrient agar (Oxoid, Thebarton, Australia). Overnight cultures (stationary phase) were prepared in nutrient broth (Oxoid, Thebarton, Australia) at 37 °C from a fresh culture. Cultures were diluted to the appropriate target inoculum (e.g., 10^3^ CFU (colony forming units)/cm^2^) with the assistance of optical density standard curves at 600 nm in a UV Visible spectrophotometer (Varian Cary 50, Varian, Mulgrave, Australia).

Toxicity and sensitivities by bacteria to coin metals were assessed by a metal disk method akin to the antibiotic disk diffusion assay [22,23]. In order to standardise the methodology across a number of different metallic currency coins, we rolled all coins down to 1 mm and punched them out to a diameter of 12 mm. This ensured that all metallic disks were of equal dimensions. A diffusion assay is based on the notion that the inhibitory compound(s) contained in the disk has to diffuse into the agar and exert its inhibitory action [23]. We placed sterile coin-metal disks in random orientation (heads or tails) on 2.5 cm Petri dishes filled with Columbia agar which were allowed to diffuse before a standard inoculum was applied and incubated for 48 h at 37 °C (see results section for details).

Cultures grown for the pre-adapted experiments were grown in liquid cultures as described above, however, the final liquid inoculum was exposed to a specific coin metal surface at a ratio of approximately 1:1 (coin surface area (cm^2^)/mL broth).

### 2.4. Extraction of Bacteria from Coins

Each sample consisted of three coins of the same denomination that were placed into 100 mL Schott bottles containing 40 mL of extraction buffer ((g/L) NaCl (10); K_2_HPO_4_ (2)), and positioned into a sonication bath (Branson 2200 Ultrasonic Cleaner with a working sonic frequency of 47 kHz ± 6%) that contained water at 25 °C. The water depth was to the shoulder of the Schott bottle. Sonication was timed for five minutes at which time the bottles were removed from the bath and placed into the Ratek mixer/incubator set at 25 °C and 80 RPM (revolutions per minute) for 30 min. After 30 min the Schott bottle was again sonicated for five minutes. The extracts were rested once more for 30 min at 25 °C in the shaker/incubator. Following extraction of bacteria from coins they were enumerated on Columbia base agar (Oxoid) and incubated at 37 °C for 48 h.

### 2.5. Extraction of Bacteria from Banknotes

Each individual banknote was placed into an individual, sterile stomacher bag after which forty ml of extraction buffer ((g/L) NaCl (10); K_2_HPO_4_ (2)) was added to each stomacher bag, which was then stomached twice for 5 min with a 30 min soak-interval [7]. Following extraction of bacteria from banknotes they were enumerated on Columbia base agar (Oxoid) and incubated at 37 °C for 48 h.

### 2.6. Scanning Electron Microscopy (SEM) of Banknote Surfaces

Small sections of banknotes were removed and coated with gold using a sputter coater (Agar Aids, Essex, UK). Banknote surfaces were visualised using a Cambridge Stereoscan 200 scanning electron microscope (Cambridge Instruments Ltd., Cambridge, UK).

## 3. Results and Discussion

### 3.1. Bacterial Survival and Adherence to Banknotes

**Figure 1:**
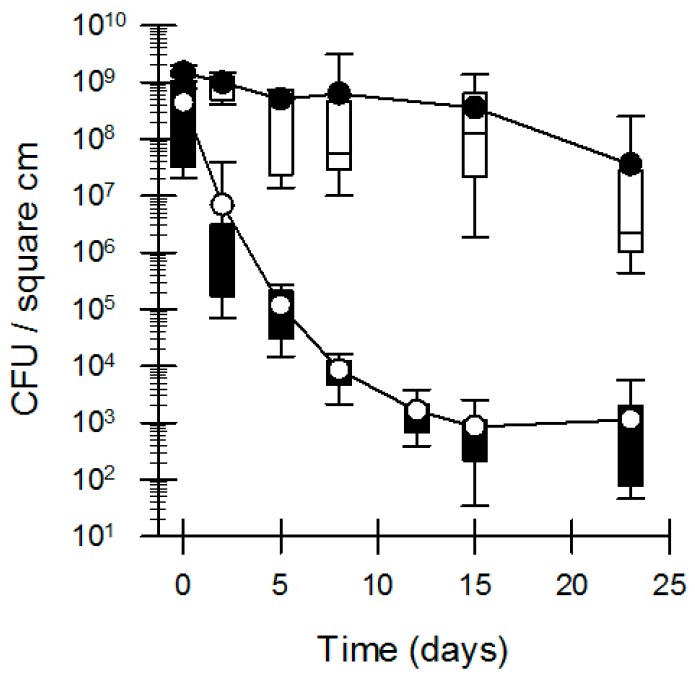
Box plot of the survival of bacteria on banknotes over time. Open boxes and closed dots: cotton-linen notes (Chinese yuan); Closed boxes and open dots: polymer (Australia dollar). Data shows recoverable bacteria after a single extraction. The bacterial inoculum (hand microbiome) in this experiment was facilitated by soaking the notes in nutrient broth in which volunteers washed their hands. The bacterial loading of the broth at the time of removing the notes from the broth was approximately 1 × 10^11^ CFU (colony forming units)/mL. The boxes represent the interquartile range (central 50%) of the data regarding the number of bacteria on banknotes analysed. The dots represent the average number of recoverable bacteria, whereas the whiskers (viz. error bars) represent either the upper or lower 25% of banknotes analysed (*n* = 30).

Past research shows that the bacterial loading on banknotes is different with regards to the substrate the notes are made of, with a noticeable lower bacterial content on polymer banknotes compared to cotton-based banknotes [7,24,25]. In order to investigate why there is a difference in the bacterial loading based on banknote substrate, we initially investigated the survival of bacteria on both cotton-based and polymer banknotes. Bacterial survival on polymer banknotes was much reduced compared to the survival on cotton-based banknotes (Figure 1). The bacterial loading on the cotton-based banknotes remained relatively steady for the first 15 days, after which there was only a minor drop in bacterial number over the following week. The bacterial loading on polymer banknotes, however, steadily dropped by almost five orders of magnitude over the first week with another ten-fold decrease in the second week. These results show that bacteria survive better on cotton-based banknotes compared to polymer banknotes.

**Figure 2:**
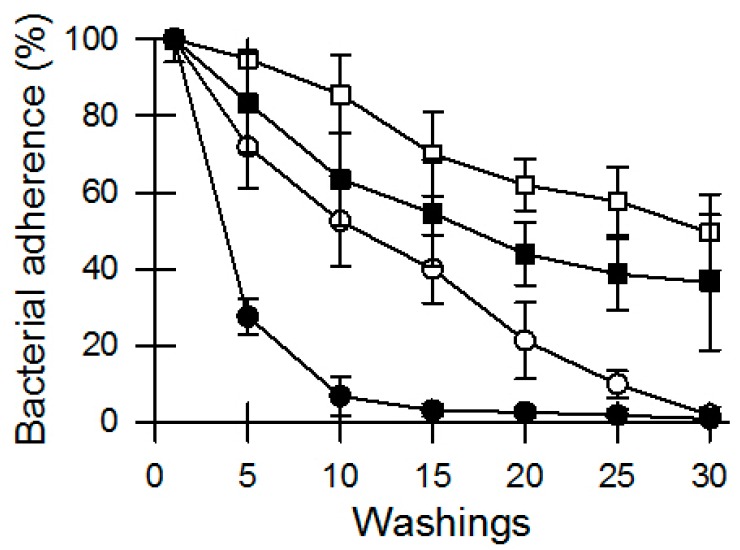
Attachment of bacteria to banknotes following multiple extractions. □ washi-style paper notes (Japanese yen); ■ cotton notes (Chinese yuan); ○ cotton-linen notes (USA dollar); and ● polymer (Australian dollar). The bacterial inoculum (hand microbiome) in this experiment was facilitated by soaking the notes in nutrient broth in which volunteers washed their hands. Data shows remaining bacteria after a series of washings in saline buffer, relative to the first washing. Data are shown are the averages of quintuplicate (*n* = 5) samples while the error bars indicate standard deviation.

Another point of differentiation with regards to bacterial loading as found on banknotes that are found in circulation could be to what extent bacteria adhere to the various substrates banknotes are made of. For this part we extended the range of available banknote substrates (microcosms). Banknotes were inoculated in the same manner as before and following overnight drying the bacteria that were dislodged from the banknotes after a series of subsequent washings in saline buffer were monitored. A relatively rapid decline in bacterial numbers over subsequent washings was taken as a sign of poor adherence of bacteria to that substrate, while a relatively slow decline in bacterial numbers was taken as a sign of resilient adherence to that substrate. Bacteria were readily washed off the polymer banknotes (Australian dollar) after 10 washings, while the washi-style paper banknotes (Japanese yen) retained over 50% of the bacteria after 30 washings (Figure 2). Cotton-based banknotes (Chinese Yuan) retained roughly 40% of the bacteria after 30 washings, while the cotton/linen-blended banknotes (USA dollar) saw a gradual decrease to zero in bacteria adhering to the notes over 30 washing (Figure 2).

**Figure 3:**
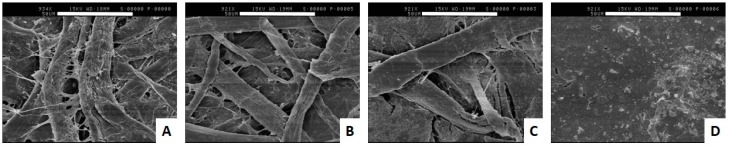
Electron micrographs (SEM) of the surface of banknotes. (**A**) Micrograph of a typical cotton-based banknote (Chinese Yuan); (**B**) micrograph of a typical washi-style paper-based banknote (Japanese Yen); (**C**) micrograph of a typical cotton-linen-based banknote (USA Dollar); and (**D**) micrograph of a typical polymer-based banknote (Australian Dollar). Scale bar represent 50 µm.

The microcosms as represented by the different banknote substrates have varying physical differences. Micro-photographs of the different substrates clearly show a very smooth surface for the plastic/polymer banknotes, while the three remaining banknote substrates are obviously fibrous (Figure 3) of varying length, width, and composition (Table 1). The combination of a smooth surface and the hydrophilic nature of the plastic banknotes are likely to be a factor in the poor adherence of bacteria to the polymer banknotes. The cotton/linen blend, cotton, and paper substrates are all of a cellulosic nature, which has a much great hydrophilic nature than plastic polymers thereby providing more moisture to potentially facilitate adherence. The fibrous nature of the cellulosic banknote substrates is most likely to be the greatest determining factor with regards to providing microcosms to which bacteria can adhere. The complexity of the three-dimensional matrix of the fibrous substrates appears to be a determining factor that supports strong adherence (i.e., cotton and washi-style paper based substrates (Figure 3A,B respectively), compared to the coarser fibres of a cotton/linen mixture (Figure 3C)).

### 3.2. Bacterial Survival and Inhibition by Coins

Coins are the most resilient and sturdiest currency items in circulation. As a rule of thumb, they represent the lower denominations and are made of metal. We investigated two of the more common metal alloys used in coining, namely: a 75/25 (copper/nickel) alloy which has a “silver” appearance and a 92/6/2 (copper/aluminium/nickel) allow which has a “gold” appearance. The coinage used in this study for the “silver” and “gold” coins were the Australian twenty cent coin and the Australian one dollar coins, respectively. The coins were inoculated by human touch, where at least five volunteers were used to handle the coins continuously for at least 15 min.

**Figure 4:**
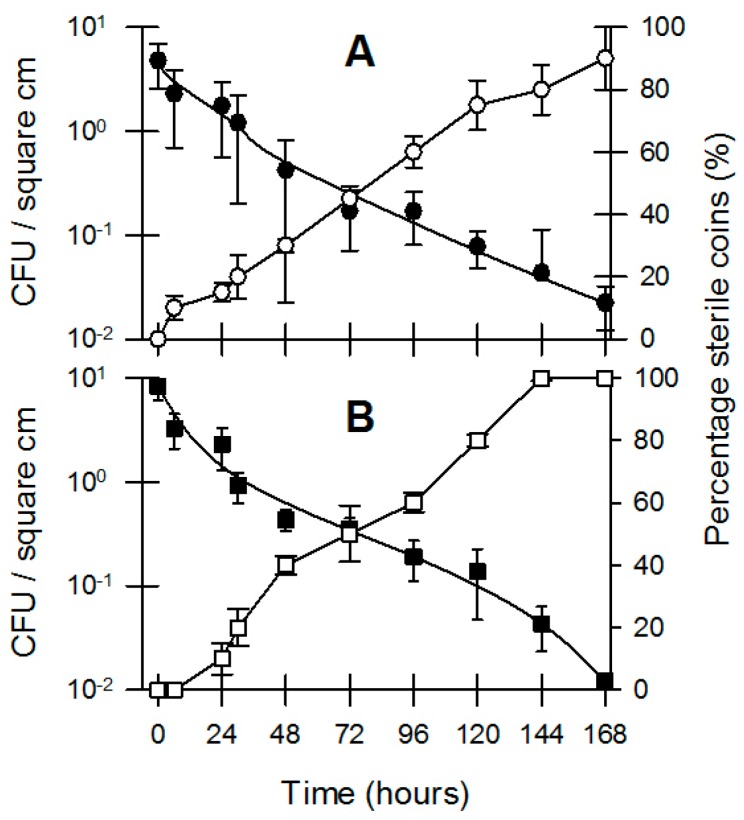
Survival of a bacterial microcosm on currency coins over time. (**A**) Australian 20 cent (silver) coins (● bacterial loading (CFU/cm^2^) on coins; ○ % sterile coins in batch of 10); (**B**) Australian one dollar (gold) coins (■ bacterial loading (CFU/cm^2^) on coins; □ % sterile coins in batch of 10). The bacterial inoculum in this experiment was facilitated by direct human contact; at least five volunteers were used to handle the currency continuously for at least 15 min (see Section 2.2 for more detail). Data shown are the average and standard deviation of recoverable bacteria after a single extraction (*n* = 10).

**Figure 5:**
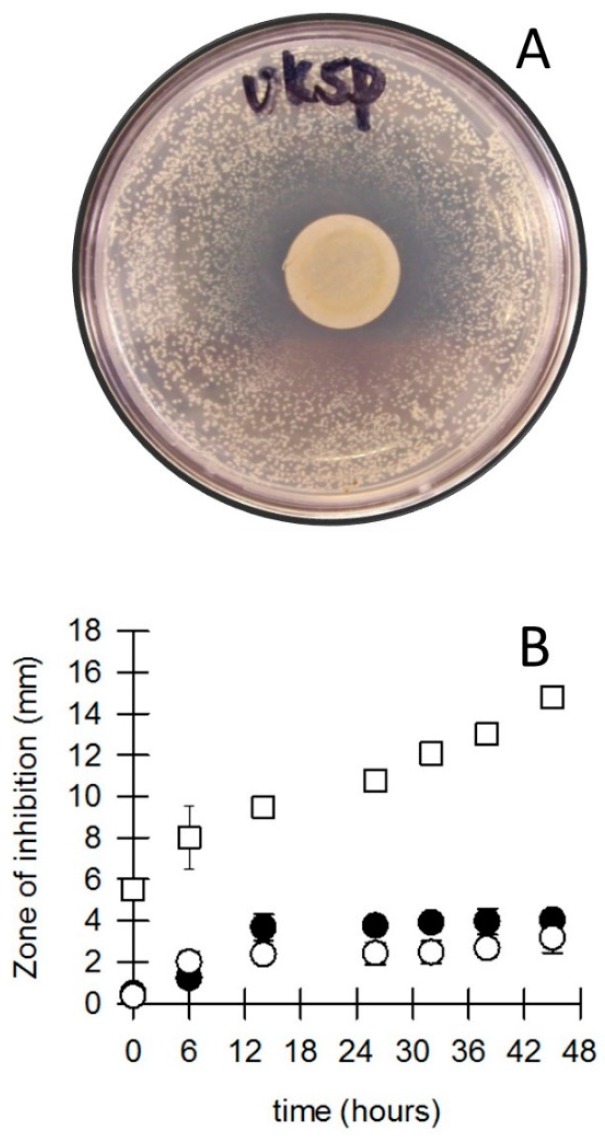
Zones of inhibition of bacteria (*Staphylococcus aureus*) due to the presence of coins. (**A**) Zone of inhibition around a 12 × 1 mm disk of a UK 5 pence coin (disk is still present in picture); (**B**) The coins were placed on the bacterial growth media at various times before the bacteria were applied. □ old Dutch bronze one cent coins; ● Australian twenty cent coins; ○ Australian one dollar coins. (*n* = 6).

Bacterial survival on both “silver” and “gold” coins was very poor. The bacterial numbers on the coins decreased steadily to near zero over one week (Figure 4), while concurrently the number of completely sterile coins increased to 100% over time (Figure 4B). The persistent bacterial die-off on “silver” and “gold” coins was similar to the observations by Jiang and Doyle [26], who showed that bacteria such as *E. coli* and Salmonella *Enteritis* rapidly died-off when exposed to coins while inert surfaces such as glass and Teflon could permit survival for much longer periods. Comparing the survival of bacteria on banknotes to the survival on coins, our results suggest that the coins might be toxic to bacteria. Toxicity and sensitivities by bacteria to metals and alloys has been widely reported [26,27,28,29,30,31]. To further investigate the notion that coins are inherently toxic to bacteria we employed a metal disk method similar to the antibiotic disk diffusion assay [22,23]. Figure 5A shows the inhibitory influence of a 12 mm disk punched from a British five pence coin. A clear zone of inhibition can be seen, followed outwards by a zone where only a limited number of very small *S. aureus* CFUs can be observed, and finally the remainder of the agar plate where the *S. aureus* CFUs grew to the same size as unexposed cells (comparative plate not shown). We first investigated the optimal time required for the inhibitory compounds from the metallic disks to diffuse into the disk before the application of a bacterial culture. For the two coin materials used in our previous experiment (Figure 4), the optimal time of metal exposure before inoculation was 15+ h after which no additional inhibition could be observed (Figure 5B), while for other coin metals (bronze) the zone of inhibition continued to increase with time. This continuation of inhibitory ability goes some way to explain the continued decrease in survival of bacteria on coins and the congruent increase in sterility of the coins.

Because different bacterial species have very divergent inhibitory responses to different inhibitory compounds [32], we used a series of pure bacterial cultures instead of a mixed culture obtained from hands to investigate variations in bacterial growth inhibition by a range of coin metals (Figure 5, Table 2). Among the coins of silver appearance are coin types of varying copper/nickel alloy mixtures, ranging from 75/25 copper/nickel (e.g., UK 5 and 10 pence coins, USA 5 cent coins, and the Australian 20 cent coins), to 84/16 copper/nickel (e.g., UK 20 pence coin), to 92/8 copper/nickel alloys (e.g., USA 10 and 25 cent coins). Among each of the copper/nickel alloy coins the same inhibitory trends were observed on *S. aureus*, *Salmonella* Typhimurium, *Escherichia coli*, *Listeria monocytogenes*, and *Cronobacter sakazakii* regardless of the country of origin of the coins (Table 2). *S.* Typhimurium, *E. coli*, and *L. monocytogenes* underwent a similar degree of inhibition to each other; however, *S. aureus* was more sensitive to the copper/nickel based coins compared to the before mentioned bacteria, while *C. sakazakii* proved to be very sensitive to copper/nickel based coins (Table 2). Both *S. aureus* and *C. sakazakii* displayed an increase in sensitivity with an increase in nickel content in the alloy. The toxicity of nickel in relation to microorganisms has been reported before [33], as well as human sensitivities to contact with nickel coins [34].

The “gold” coloured coins (copper/aluminium/nickel alloy as represented by the New Zealand and Australian one dollar coins) presented quite a different degree of inhibition (Table 2—NZ 1$). *S. Typhimurium*, *L. monocytogenes*, and *C. sakazakii* showed no growth inhibition with CFUs growing up to the edge of the coin disks. However, *S. aureus* and *E. coli* revealed a marked level of growth inhibition due to the presence of the gold coins’ disks (Table 2). The British one pound coin is an alloy of copper, zinc, and nickel, which caused growth inhibition with regards to *S.* Typhimurium, *E. coli*, and *L. monocytogenes*, and a more substantial inhibition of growth towards *S. aureus* and *C. sakazakii*.

There is currently a trend among the world’s mints to produce coins more economically, which has seen a release of stainless steel and plated coins in favour of the more expensive alloys. Completely stainless steel fabricated coins (as represented by the Mexican 10 centavos coin) imparted a similar degree of growth inhibition as was observed for the British one pound coin, however, the stainless steel coins did not inhibit *C. sakazakii*. By far the most inhibitory coins were the modern American one-penny (Table 2). The American one-penny coin represents the newer style of coins with a core of one metal and plated with a second metal: the American penny consists of a copper-plated zinc core. The very large degree of growth inhibition displayed by the American penny is quite remarkable, but could well have been an artefact of our treatments of the coins. As mentioned earlier, we rolled out the coins to disks of an even thickness, which were then punched out to a standard diameter. The fact that both the core metal and the plating metal were exposed to the agar might have caused a galvanic corrosion in which an altered rate of diffusion occurs and/or toxic byproducts are produced [35]. However, galvanic influences do not apply to all plated coins. The modern New Zealand 10 and 50 cent coins are made of a steel core that are nickel plated and these coins did not exhibit the same magnitude of growth inhibition (Table 2) as was seen by the copper plated zinc coins.

**Figure 6:**
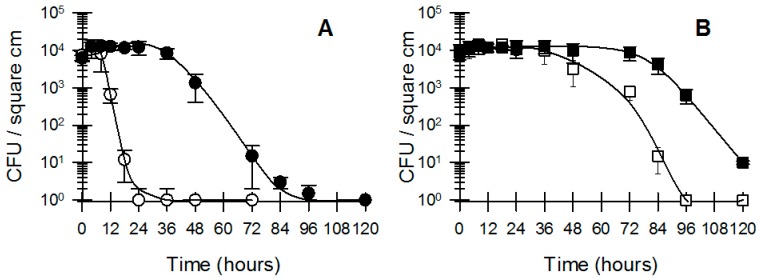
Effect of pre-exposure of bacterial parent cultures to coin metal (Australian $1) prior to survival on the same coinage. (**A**) *L. monocytogenes*; ○ non-exposed culture; ● pre-exposed culture; (**B**) *E. coli*; □ non-exposed culture; ■ pre-exposed culture. The bacterial inoculum in this experiment was facilitated by introducing a fixed aliquot of bacterial suspension to one side (heads only) of sterile coins. Data shown are the average and standard deviation of recoverable bacteria after a single extraction (*n* = 6).

While it is obvious that a wide range of bacteria are present on the surfaces of coins in varying qualities [3,6,9,21,36,37,38,39,40], our previous data (Figure 5 and Table 2) show that many bacteria are inhibited by exposure to coins. In order to shed light on the fact that bacteria are still present on coins that are in circulation, we investigated whether bacteria would adapt to their environment when they were pre-exposed to coin metal. We compared two inocula of pure cultures of *E. coli* and *L. monocytogenes* for their survival on Australian $1 coins. The first inoculum was grown in standard Nutrient Broth, while the second inoculum was grown in Nutrient Broth in the presence of sterile AU $1 coins at a 1:1 surface-area: volume (cm^2^/mL) ratio. Inocula that were grown in the presence of sterile AU $1 coins remained viable for longer compared to inocula grown in standard Nutrient Broth (Figure 6). Our data show that different bacteria will survive longer when exposed to coin metal and that the survival of *L. monocytogenes* was greater when pre-exposed to coins as a stressor compared to *E. coli* (Figure 6A,B). These results go some way to explain the survival of bacteria on coins over extended periods of time. Our data show that coins are of an inherent inhibitory nature with regards to bacterial survival; however, the data also show that bacteria have the ability to adapt to coin metals in their environment and improve their longevity. In a final experiment we screened Australian $1 coins and 20 cent coins that had not been handled by humans for over four years for the presence of bacteria by means of ATP (adenosine triphosphate) bioluminescence and enrichment followed by spot plating. We used coins that were “as good as new”, and coins that were so dirty and damaged that they were taken out of circulation by the Australian mint. All coins returned extremely low levels of ATP bioluminescence, while the “as good as new” coins were deemed to be sterile based on the enrichment and spot plating procedure. However, 40% of the very dirty coins ($1 and 20 cent) showed the presence of Gram positive, sporeforming bacteria as the surviving microbiome.

## 4. Conclusions

The microbiome associated with human hands in association with currency has provided an insight into the factors that influence the presence of bacteria on banknotes and coins (the microcosms). The bacterial presence on banknotes is very much influenced by the material the banknotes are made of. Banknotes made of material with coarse fibres provide greater adherence, while banknotes with a smooth surface provide poor bacterial adherence. Similarly, bacteria survive better on banknotes made with natural fibres compared to poor survival on plastic banknotes. It does not appear that banknotes have a toxic effect on the bacterial microbiome; instead, it appears that banknotes made of hydrophobic polymers provide an inert microcosm which does not provide suitable habitat that supports long-term bacterial survival. The bacterial presence on coins appears to be far more transient compared to their presence on banknotes. The bacterial microbiome associated with human hands survives poorly on coins, which appears to be due to a direct toxic influence from the coins on the bacteria. However, bacteria have the ability to adapt to the presence of coin metals in their environment and improve their longevity by the time they have adjusted to the presence of coin metals.

The findings presented here go some way to explain the persistence of bacteria on both coins and banknotes. It will need to be recognised that the bacterial microbiome on coins and banknotes can only be representative of the previous exposure of the currency microcosm to the environmental inputs such as handling of currency by humans.

## Figures and Tables

**Table 1:** Properties of Natural Fibres present in Banknotes.

Notes & Composition	Cotton *Gossypium* spp	Linen *Linum usitatissimum*	Mitsumata *Edgeworthia papyrifera*	Abaca *Musa textilis*
Chinese and UK notes	100%			
USA notes	~75%	~25%		
Japanese notes			~67%	~33%
Fibre length (mm)	10–40	9–70	2–4	2–12
Fibre width (μm)	12–38	5–40	4–20	15–40
Cellulose	74–98	64.1	50	60
Hemicellulose	1–11	17	20	21
Lignin	1–16	2	4	12

Adapted from: [19,20,21].

**Table 2:** Inhibitory influence of coin disks (12 × 1 mm) on the growth of a range of bacteria as represented by zones of inhibition in average and standard deviation, and mm radius (*n* = 12).

Coinage *			Zone of Inhibition (mm)				
Currency		→ Bacteria → →	*S. aureus*	*S. Typhimurium*	*E. coli*	*L. monocytogenes*	*C. sakazakii*
	↓ Make-up ↓						
USA 1c	Copper plated zinc		37.1 (3.7)	30.9 (6.9)	34.3 (7.1)	37.3 (9.0)	45.5 (3.8)
UK £1	70% copper, 24.5% zinc, 5.5% nickel		13.8 (1.7)	3.9 (0.9)	1.3 (0.8)	2.8 (1.1)	8.9 (1.1)
AU $1; NZ $1	92% copper, 6% aluminium, 2% nickel		4.9 (0.4)	3.8 (1.1)	2.5 (0.7)	2.1 (0.7)	7.6 (1.6)
MEX 10c	100% stainless steel		12.2 (2.9)	2.5 (0.6)	1.7 (0.6)	2.0 (0.7)	0.0
NZ 50c	Nickel plated steel		2.6 (0.5)	4.2 (0.8)	4.6 (0.9)	3.9 (1.2)	5.6 (0.56
UK 10p; USA 5c; AU 20c	75% copper, 25% nickel		8.8 (2.3)	2.3 (0.6)	2.3 (1.1)	1.5 (1.1)	18.4 (1.4)
UK 20p	84% copper, 16% nickel		7.9 (2.1)	2.2 (1.0)	2.0 (0.6)	1.8 (0.9)	16.4 (1.7)
USA 10c; 25c	92% copper, 8% nickel		6.6 (1.4)	1.8 (0.3)	2.3 (1.1)	1.9 (0.4)	13.1 (1.6)

* In order to standardise the methodology across a number of different metallic currency coins we rolled all coins down to a 1 mm thickness and punched them out to create disks with a diameter of 12 mm.
